# 1*H*-Imidazol-4(5*H*)-ones and thiazol-4(5*H*)-ones as emerging pronucleophiles in asymmetric catalysis

**DOI:** 10.3762/bjoc.12.90

**Published:** 2016-05-09

**Authors:** Antonia Mielgo, Claudio Palomo

**Affiliations:** 1 Departamento de Química Orgánica I, Facultad de Química, Universidad del País Vasco, Apdo. 1072, 20080 San Sebastián, Spain

**Keywords:** asymmetric catalysis, bifunctional catalysts, 1*H*-imidazol-4(5*H*)-ones, pronucleophiles, thiazol-4(5*H*)-ones

## Abstract

Asymmetric catalysis represents a very powerful tool for the synthesis of enantiopure compounds. In this context the main focus has been directed not only to the search for new efficient chiral catalysts, but also to the development of efficient pronucleophiles. This review highlights the utility and first examples of 1*H*-imidazol-4(5*H*)-ones and thiazol-4(5*H*)-ones as pronucleophiles in catalytic asymmetric reactions.

## Introduction

**Figure 1:**
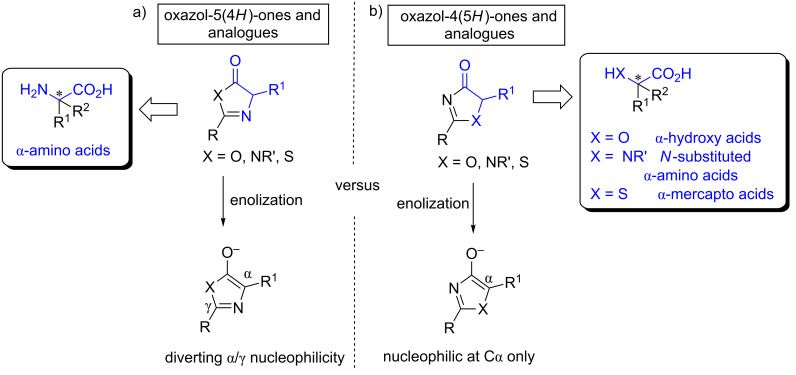
Some α-substituted heterocycles for asymmetric catalysis, their reactivity patterns against enolization, and resulting adducts after catalytic reaction and hydrolysis.

Asymmetric catalysis [1–3] constitutes a very powerful tool for the preparation of enantiomerically pure compounds [4]. Recent efforts in the field have been devoted to the development of new efficient chiral catalysts, both metal catalysts and organocatalysts, together with the search for appropriate (pro)nucleophiles and/or electrophiles. In this context, the enantioselective construction of tetrasubstituted stereocenters is another challenge [5–13]. Regarding reactions which involve proton transfer events, soft enolization [14–16] constitutes an efficient tool for the deprotonation of some carbonyl compounds [17–18]. In these cases, a relatively weak amine is generally used to reversibly deprotonate a relatively acidic substrate; however, to date, the carbonyl components for these reactions are mostly restricted to 1,3-diones, β-ketoesters, malonates, α-cyanoacetates, 3-substitued oxindoles and related systems. Generally, enolizable esters or carboxylic acid derivatives have been challenging in this strategy, because their p*K*a values (approximately 19 in DMSO) [19] are much higher, one exception being thioesters [20–21] and pyrazoleamides [22]. As an alternative, some specific heterocycles have been proposed as carboxylic acid surrogates. Examples of this type of heterocycles are oxazol-5-(4*H*)-ones (or azlactones) and their analogues (Figure 1a) and oxazol-4-(5*H*)-ones and their thiazolone and imidazolone analogues (Figure 1b).

These heterocycles show very interesting characteristics: i) easy deprotonation under soft enolization conditions (aromatic enolate formation); ii) the geometry of the resulting starting enolate or equivalent is fixed due to their cyclic nature, thus facilitating the control of the stereoselectivity; iii) they are substituted at the α-position of the carbonyl and therefore, after reaction with an electrophile, a tetrasubstituted stereocenter is created and, iv) the resulting adducts can be easily hydrolyzed to provide carboxylic acids or their derivatives carrying different functionalities.

**Figure 2:**
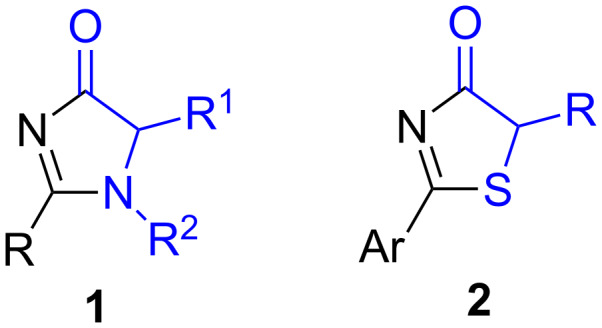
1*H*-Imidazol-4(5*H*)-ones **1** and thiazol-4(5*H*)-ones **2**.

The most common pronucleophiles of this type are oxazol-5-(4*H*)-ones or azlactones (Figure 1a, X = O), which have been thoroughly investigated and reviewed [23–25]. On the other hand, since the pionnering work by Trost in 2004 [26], several examples of the utility of the structurally related oxazol-4(5*H*)-ones (Figure 1b, X = O) have also been published, which involve mainly Michael additions (to enones [27–28], nitroalkenes [29–30], alkynones [31–33] and vinyl sulfones [34]), γ-additions to allenic ketones and esters [35–36], 1,6-additions to conjugated dienones [37], aldol/Mannich reactions [26,38–40], α-sulfenylation reactions [41] and alkylations [42–43]. More recently the nitrogen (1*H*-imidazol-4(5*H*)-ones, Figure 1b, X = NR’) and sulfur (thiazol-4(5*H*)-ones, Figure 1b, X = S) analogues of these oxazol-4(5*H*)-ones have been demonstrated to be very interesting templates in asymmetric catalysis. In these cases the hydrolysis of the adducts coming from an asymmetric reaction can provide enantioenriched α-hydroxy acids (X = O), *N*-substituted α-amino acids (X = NR’) or α-mercapto acids (X = S) depending on the nature of the starting heterocycle. This review describes the first examples and applications of 1*H*-imidazol-4(5*H*)-ones **1** and thiazol-4(5*H*)-ones **2** (Figure 2) as emerging pronucleophiles in asymmetric catalytic reactions.

## Review

### 1 1*H*-Imidazol-4(5*H*)-ones **1**

α,α-Disubstituted (quaternary) amino acids are compounds and/or scaffolds of demanding continuous interest [44] and many stereoselective protocols have been developed for their syntheses [44–50]. In this field, particularly interesting are *N*-substituted derivatives, which are potentially good therapeutical candidates because of their high lipophilicity and membrane permeability [44,51]. A major catalytic entry to α,α-disubstituted (quaternary) amino acids is the α-functionalization of an appropriate template as, for instance, an α-imino ester or lactone, followed by hydrolysis [49,52–53]; but, however, very few of them provide *N*-substituted derivatives directly [54]. In this context, 1*H*-imidazol-4(5*H*)-ones **1** have been recently introduced as novel nucleophilic α-amino acid equivalents for the catalytic and asymmetric synthesis of *N*-alkyl, *N*-aryl and *N*-allyl α,α-disubstituted amino acids [55]. The main advantages of these pronucleophiles over the previous known templates are: i) the NR group can be installed in the heterocycle previous to the asymmetric reaction, ii) they are easily deprotonated under soft enolization conditions (aromatic enolate formation), and iii) unlike azlactones and analogous they do not show the Cα/Cγ selectivity problem [56–58].

#### 1.1 Synthesis of 1*H*-imidazol-4(5*H*)-ones **1**

**Scheme 1:**
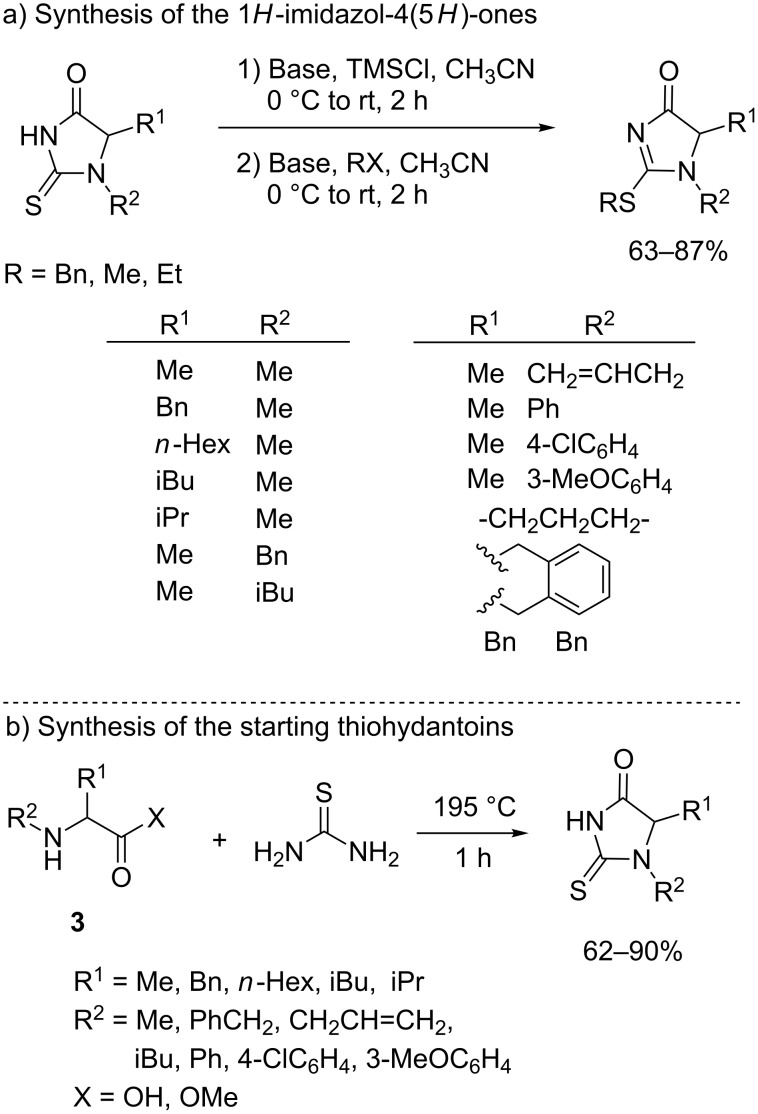
a) Synthesis of 2-thio-1*H*-imidazol-4(5*H*)-ones [55] and b) preparation of the starting thiohydantoins [59].

1*H*-Imidazol-4(5*H*)-ones **1** (R = SBn) are prepared by *S*-alkylation of the corresponding thiohydantoins [55,59] (Scheme 1a) prior trimethylsilyl enol ether formation which is necessary to avoid *O*-alkylation. The starting thiohydantoins, in turn, are obtained by heating a mixture of the corresponding *N*-substituted amino acid or its methyl ester **3** and thiourea at 195 °C [59] (Scheme 1b). Following this protocol different 1*H*-Imidazol-4(5*H*)-ones have been prepared in good yields.

#### 1.2 1*H*-Imidazol-4(5*H*)-ones **1** as pronucleophiles in organocatalyzed Michael addition reactions

2-Thio-1*H*-imidazol-4(5*H*)-ones **1** (R = SBn) have been reported to be effective equivalents of *N*-subtituted (alkyl, aryl, allyl) α-amino acids in conjugate addition reactions to both, nitroalkenes and α-silyloxy enones as Michael acceptors in reactions promoted by bifunctional Brønsted bases.

**Scheme 2:**
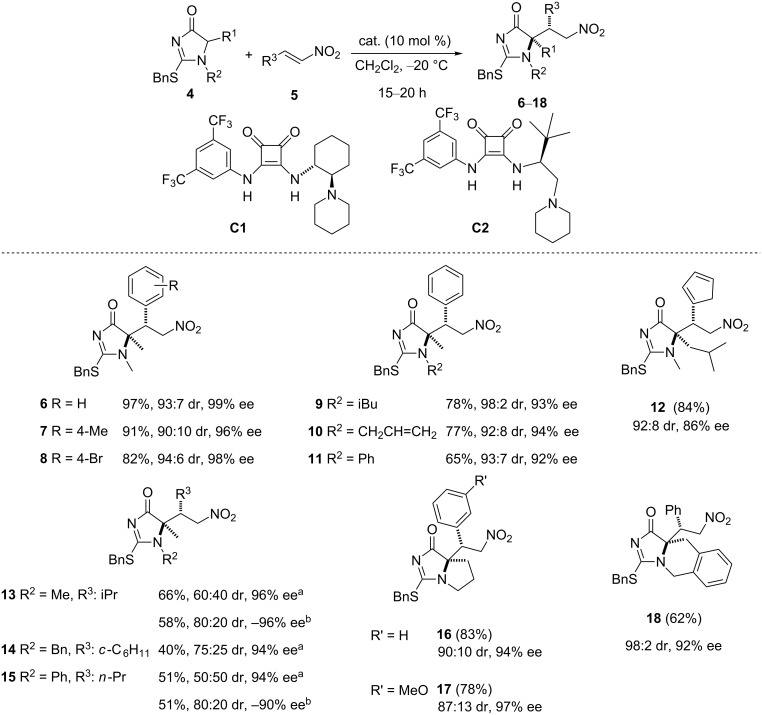
Selected examples of the Michael addition of 2-thio-1*H*-imidazol-4(5*H*)-ones to nitroalkenes [55]. ^a^Reactions run at 50 °C in CH_2_Cl_2_. ^b^Using catalyst **C2**.

**1.2.1 Nitroalkenes as acceptors.** Investigation of the base-catalyzed Michael addition reaction of 2-thio-1*H*-imidazol-4(5*H*)-ones **4** to nitroalkenes **5** [55] revealed that cinchona alkaloids such as quinine, (DHQ)_2_Pyr or even thiourea tertiary amine catalysts were not efficient in terms of stereoselectivity. However, good results regarding both, yield and stereocontrol, were observed when the Rawal catalyst **C1** [60–61] was employed (Scheme 2).

Under the optimized conditions the reaction, as shown in Scheme 2, worked equally well regardless of the electronic character of the β-aryl substituent of nitroalkene **5** (compounds **6**–**8**). Different substituents either at the nitrogen atom of the starting imidazolone (compounds **9**–**11**) or at the 5-position (compound **12**) of the ring are also well tolerated. Reactions with β-alkyl nitroolefins in the presence of **C1** (Scheme 2, compounds **13**–**15**) proceeded with poor diastereocontrol but the results were improved by changing to catalyst **C2** [62]. Nonetheless, in both cases the enantioselectivity for the major diastereomer was excellent. Finally, and starting from the corresponding bicyclic imidazolones, quaternary proline and related derivatives (**16**–**18**), which are difficult to obtain through established catalytic methodologies [63–65], can also be efficiently synthesized.

**Scheme 3:**
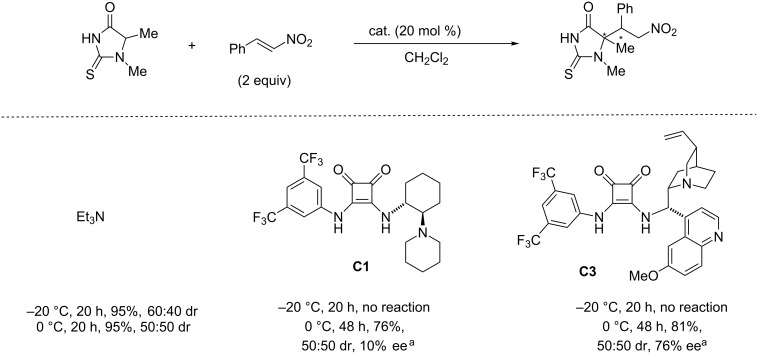
Michael addition of thiohydantoins to nitrostyrene assisted by Et_3_N and catalysts **C1** and **C3**. ^a^Absolute configuration not determined.

On the other hand, it is worthy of note that thiohydantoins, which are structurally related to 2-thio-1*H*-imidazol-4(5*H*)-ones **1**, have been demonstrated to be either less reactive and/or less stereoselective in their addition reaction to nitrostyrene thus affording the corresponding Michael adducts with no diastereoselectivity and/or poor enantioselectivity (Scheme 3).

**Scheme 4:**
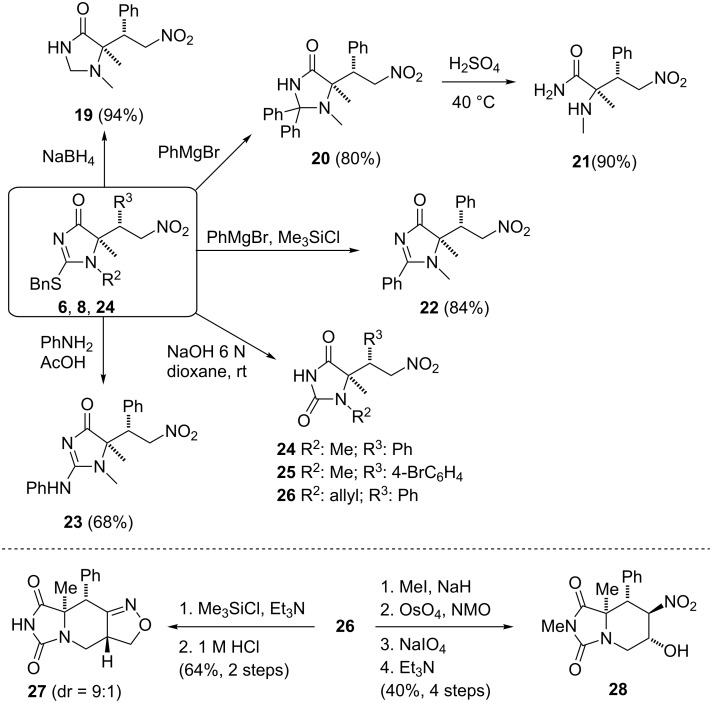
Elaboration of the Michael adducts coming from the Michael addition to nitroalkenes [55].

Useful applications of the Michael adducts coming from the Michael addition of imidazolones to nitroalkenes are shown by the transformations depicted in Scheme 4. Thus, nucleophilic displacement of the thioether group gives access to various types of heterocycles of interest in medicinal chemistry [66–67] (i.e., imidazolidinones **19** and **20**, 2-arylimidazolone **22**, 2-aminoimidazolone **23** and hydantoins **24**–**26**). On the other hand, acid hydrolysis of these adducts efficiently affords *N*-alkylamino acid derivatives, as for instance the *N*-methylamino amide **21**. Additionally, from the common adduct **26**, functionalized polycyclic hydantoins of type **27** and **28** can also be synthesized.

**Figure 3:**
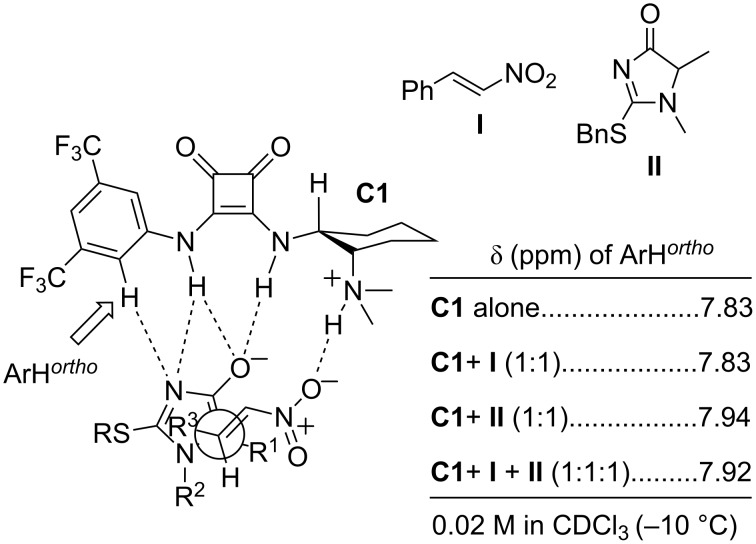
Proposed model for the Michael addition of 1*H*-imidazol4-(5*H*)-ones and selected ^1^H NMR data which support it [55].

The authors proposed an heuristic model (Figure 3) to account for the experimentally encountered selectivity where the catalyst is proposed to act in a bifunctional way. In this proposal the imidazolone would be coordinated to the two NH bonds of the squaramide and the ortho-ArH in **C1**, whilst the nitroalkene would form a hydrogen bond by coordination to the protonated tertiary amine through the oxygen of the nitro group. This assumption was supported by the fact that the chemical shift of the *ortho*-ArH in **C1** varies considerably after the addition of 1 equivalent of imidazolone to a solution of **C1** in CDCl_3_, whereas it doesn’t change after the addition of nitrostyrene.

**1.2.2 α-Silyloxyenones as acceptors.** Among Michael acceptors, simple α,β-unsaturated esters and amides still are challenging substrates in direct Michael additions and have only been employed in few successful Michael reactions, mainly due to their inherent lower reactivity and the limitations associated to the activation/coordination of these compounds to a suitable chiral catalyst. Although recently it has been shown that the problem of this low reactivity may be addressed through the development of Brønsted base catalysts with increased basicity [68], most efforts still focus on the development of unsaturated ester/amide surrogates [69–71], which involve α,β-unsaturated imides, *N*-acyl heterocycles, α-oxophosphonates, α-ketoesters, 3-methyl-4-nitro-5-alkenylisoxazoles and α’-hydroxyenones. These latter substrates have proven to be very efficient platforms for bidentate coordination in metal catalysis and good precursors of carboxylic acids, ketones and aldehydes upon oxidative cleavage of the keto/diol moiety [72]. More recently, a comprehensive study on the first evidence of the utility of these acceptors in organocatalysis has been published [73]. This study shows that α’-oxyenones are very efficient key enoate equivalents in Brønsted base-catalyzed asymmetric conjugate addition of a range of soft nucleophiles such as α-substituted oxindoles, cyanoesters, oxazolones, azlactones and thiazolones to afford the corresponding tetrasubstituted Michael adducts in high diastereo- and enantioselectivity. The efficiency of the previous 2-thio-imidazol-4(5*H*)-ones as pronucleophiles in Michael reactions has also been corroborated in the addition to these α-silyloxyenones as Michael acceptors [55].

**Scheme 5:**
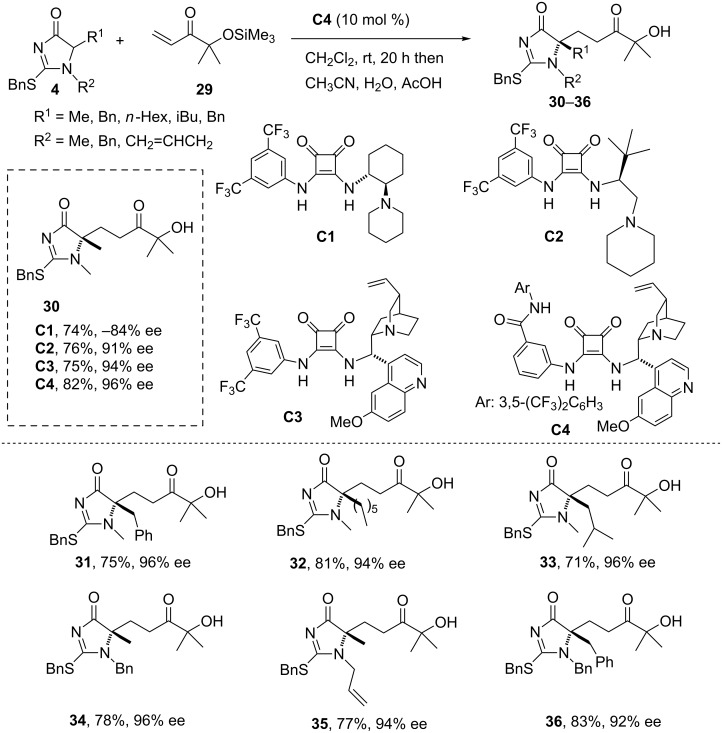
Michael addition 2-thio-1*H*-imidazol-4(5*H*)-ones to the α-silyloxyenone **29** [55].

First attempts to carry out the Michael addition reaction of imidazolones to simple unsaturated esters and ketones revealed that whilst these reactions worked sluggish, they proceeded efficiently when α-silyloxyenones were used as Michael acceptors. In these cases and, under the conditions shown in Scheme 5, reaction of the 1,5-dimethylimidazolone **4** (R^1^ = R^2^ = Me) in the presence of catalyst **C1** afforded, after desilylation, the Michael adduct **30** in good yield (74%) but in moderate enantioselectivity (−84%). Improved selectivity (91%) was observed using **C2** in the reaction with **4** (R ^1^= R^2^ = Me) and even better enantioselectivity was obtained with catalysts **C3** (94%) and **C4** (96%). Under these optimized conditions a survey of imidazolones reacted with enone **29** to produce adducts with yields within the range 71–83% and with very high enantioselectivity (Scheme 5).

**Scheme 6:**
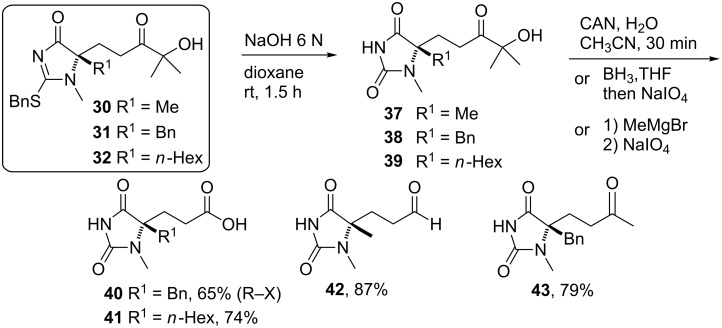
Elaboration of the Michael adducts coming from the Michael addition to nitroolefins [55].

The Michael adducts can also be transformed into different derivatives (Scheme 6), particularly into hydantoins **37**–**43** after hydrolysis of the corresponding adducts **30**–**32** [55]. Oxidative elaboration of the ketol unit in these compounds provides the corresponding carboxylic acids **40**/**41**, the aldehyde **42** and the ketone **43**. Therefore this methodology facilitates a novel entry to functionalized 5,5-disubstituted hydantoins, a well-recognized scaffold for drug discovery.

### 2 Thiazol-4(5*H*)-ones **2**

**Scheme 7:**
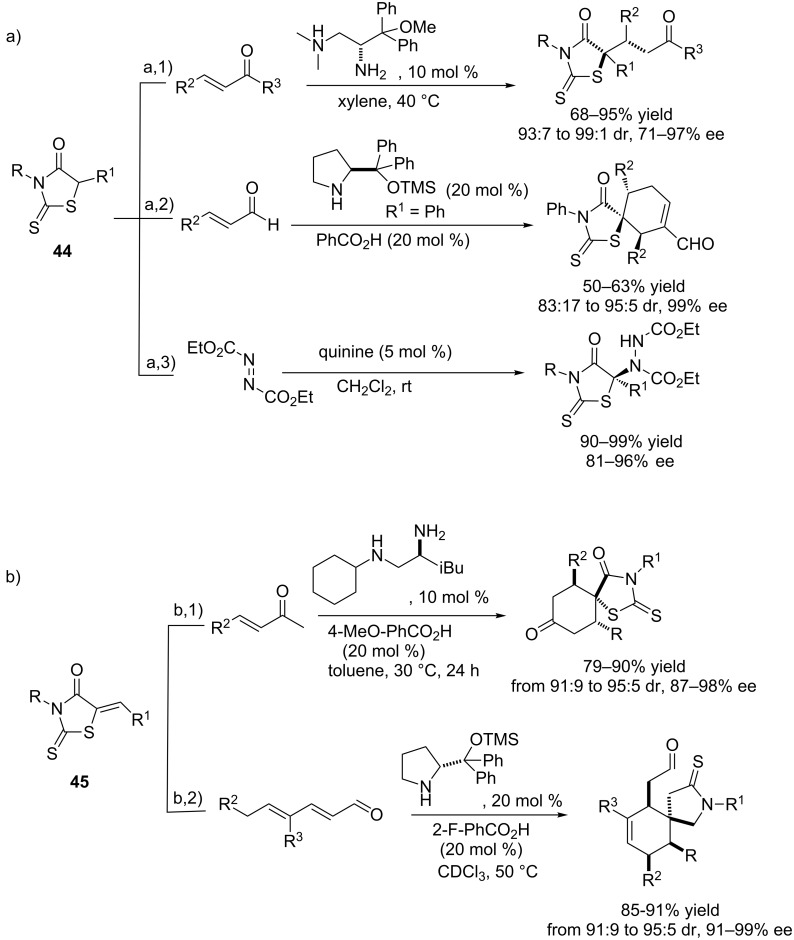
Rhodanines in asymmetric catalytic reactions: a) Reaction with rhodanines of type **44** [78–80]; b) reactions with rhodanines of type **45** [81–82].

Thiazol-4(5*H*)-ones **2**, which exhibit interesting applications in medicinal and pharmaceutical areas [74–76], can be easily synthesized and can act as (pro)nucleophiles in asymmetric catalytic reactions. In 2011, Weib and Beckert et al. reported a ^1^H NMR study of these compounds in solution that shows that they exist in equilibrium between two tautomeric forms [77], and therefore this could facilitate deprotonation at the 5-position to further react with various electrophiles. However, thiazol-4(5*H*)-ones have been until now rarely used in asymmetric catalysis, and only very recently four interesting examples describing their applications in this realm have been reported. On the other hand, rhodanines **44** and **45** (Scheme 7), heterocycles structurally related to thiazol-4(5*H*)-ones **2**, have also been very scarcely used as pronucleophiles in asymmetric catalysis. Only few examples have been reported, some of them involving the use of rhodanines of type **44** (Scheme 7a), which act directly as pronucleophiles against enones [78] (Scheme 7a,1), enals [79] (a,2) and azodicarboxylates [80] (a,3). In two other examples (Scheme 7b), however, rhodanines of type **45** have been employed to produce spirocyclic compounds. The first case is an enamine/Michael tandem reaction to unsaturated enones [81] (Scheme 7b,1) and the second one is the Diels–Alder reaction with 2,4-dienals which occurs via trienamine formation [82] (Scheme 7b,2).

#### 2.1 Synthesis of thiazol-4(5*H*)-ones **2**

Thiazol-4(5*H*)-ones **2** can be easily prepared starting from the corresponding α-mercaptocarboxylic acid and nitrile. Treatment of both with triethylamine in refluxing ethanol [83] provides the expected thiazol-4(5*H*)-ones as yellow/green solids in good yields. The starting α-mercaptocarboxylic acids can be prepared by reaction of the corresponding α-bromo derivatives with potassium thioacetate followed by treatment with ammonia in methanol [84].

#### 2.2 Thiazol-4(5*H*)-ones **2** as pronucleophiles in asymmetric catalytic reactions

The potential of thiazol-4(5*H*)-ones as pronucleophiles in asymmetric catalytic reactions has been investigated in the Michael addition reaction to nitroalkenes and α-silyloxyenones, phosphine-catalyzed γ-addition to allenoates and alkynoates, α-amination reactions and iridium-catalyzed allylic substitution reactions.

**Scheme 8:**
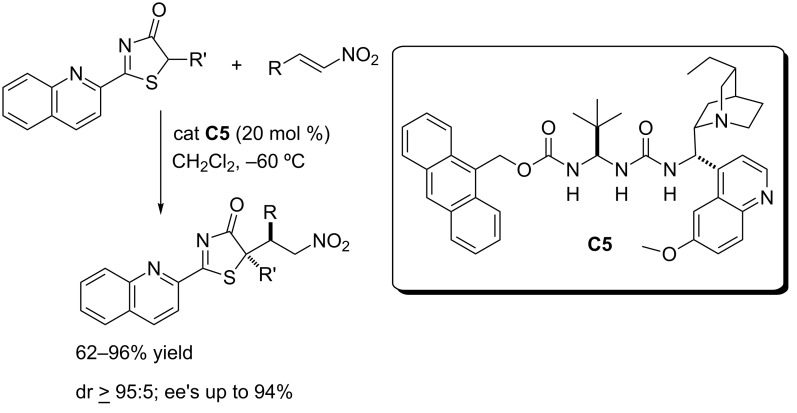
Michael addition of thiazol-4(5*H*)-ones to nitroolefins promoted by the ureidopeptide-like bifunctional Brønsted base catalyst **C5** [85].

**2.2.1 Michael addition reactions, nitroalkenes as acceptors.** The first example of the utility of the thiazol-4(5*H*)-ones **2** as pronucleophiles in asymmetric catalysis was reported in 2013 in the Michael addition to nitroalkenes catalyzed by the bifunctional ureidopeptide-like Brønsted base **C5** (Scheme 8) [85].

**Figure 4:**
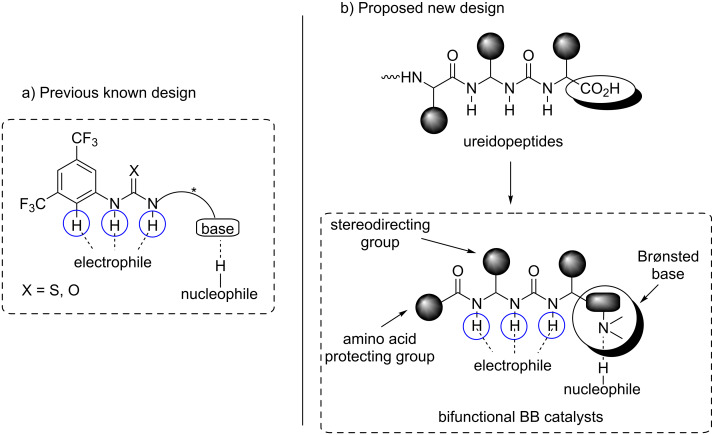
Ureidopeptide-like Brønsted bases: catalyst design. a) Previous known design. b) Proposed new design. BB: Brønsted base [85].

This catalyst belongs to a new subclass of bifunctional Brønsted bases, which was developed on the basis of Takemoto’s model [86]. This model is featured by three different moieties: a basic site, a urea (thiourea) function and a 3,5-bis(trifluoromethyl)phenyl group, all three elements being necessary for catalyst activity (Figure 4a) [87–89]. In 2010 Zhong proposed that the presence of the ortho C–H bond of the aryl group could be the key for success because it could participate together with the thiourea function in the activation of the electrophile [90]. This proposal was in 2012 supported by Schreiner [91] after an exhaustive study based on NMR/IR spectroscopy, mass spectrometry and theoretical DFT calculations. By taking into account these characteristics, the authors considered that the combination of ureidopetide’s structure, which have been recognized by their ability to develop hydrogen bond interactions [92–95] with a Brønsted base could provide a new family of potentially efficient bifunctional catalysts (Figure 4b). The main features of these new catalysts are the presence of a urea moiety together with a *N*,*N*-diacylaminal unit, both in close proximity to an additional stereodirecting group.

**Scheme 9:**
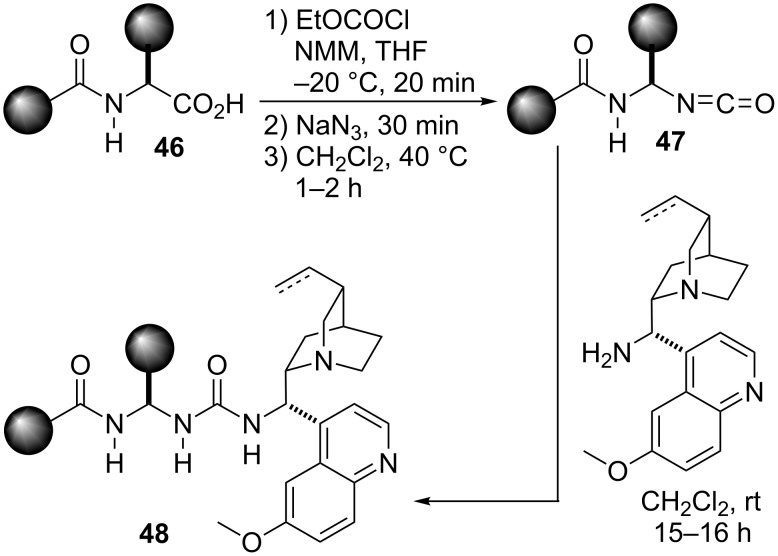
Ureidopeptide-like Brønsted base bifunctional catalyst preparation. NMM = *N*-methylmorpholine, THF = tetrahydrofuran [85].

**Scheme 10:**
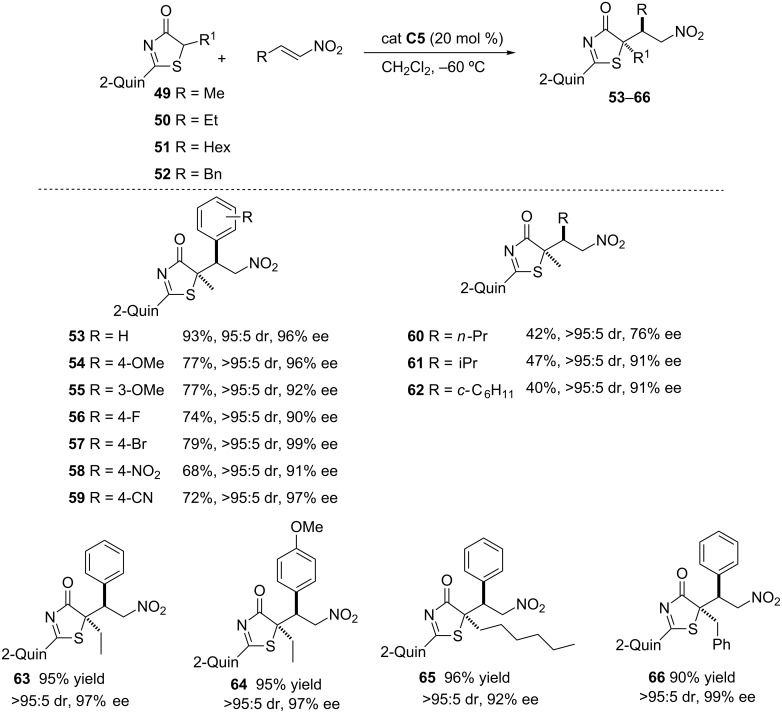
Selected examples of the Michael addition of thiazolones to different nitroolefins promoted by catalyst **C5** [85].

Following this design and starting from the corresponding *N*-protected α-amino acid **46** different catalysts were easily synthesized by the reaction of the respective intermediate isocyanates **47** [93] with 9-*epi*-9-amino-9-deoxyquinine or 9-*epi*-9-amino-9-deoxyhydroquinine (Scheme 9). These catalysts were tested in the reaction between 5-methylthiazolone **49** and nitrostyrene (Scheme 10) and after optimization catalyst **C5** was found to be the optimal for this transformation.

A representative selection of nitroalkenes was then evaluated in the presence of catalyst **C5** and, as the data in Scheme 10 show, those bearing β-aryl substituents with either electron-donating or electron-withdrawing groups produced the corresponding adducts in good enantio- and diastereoselectivities (compounds **53**–**59**). Even the most problematic β-alkyl nitroalkenes worked well affording the adducts in good stereoselectivity albeit in poor yields (Scheme 10, compounds **60**–**62**). The reaction was equally efficient with thiazolones carrying short and large alkyl chains (Scheme 10, compounds **63**–**66**).

**Scheme 11:**
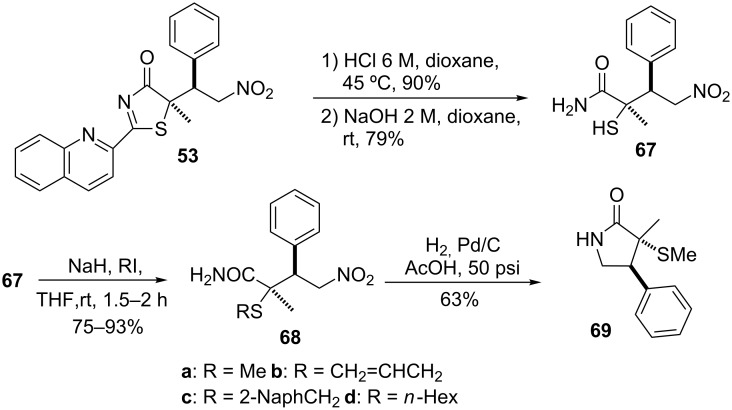
Elaboration of the Michael adducts to α,α-disubstituted α-mercaptocarboxylic acid derivatives [85].

An aspect of practical interest of this methodology is that whilst the majority of the procedures for the preparation of organosulfur compounds render thioether derivatives [96–97], this approach provides products with a free thiol group such as the α-mercaptocarboxylic acid derivative **67** (Scheme 11). Additionally, these mercapto derivatives can also be *S*-alkylated by treatment with the corresponding halide in the presence of sodium hydride, giving thus access to thioether derivatives of type **68**, which can also be converted into γ-lactams such as **69**.

**Scheme 12:**
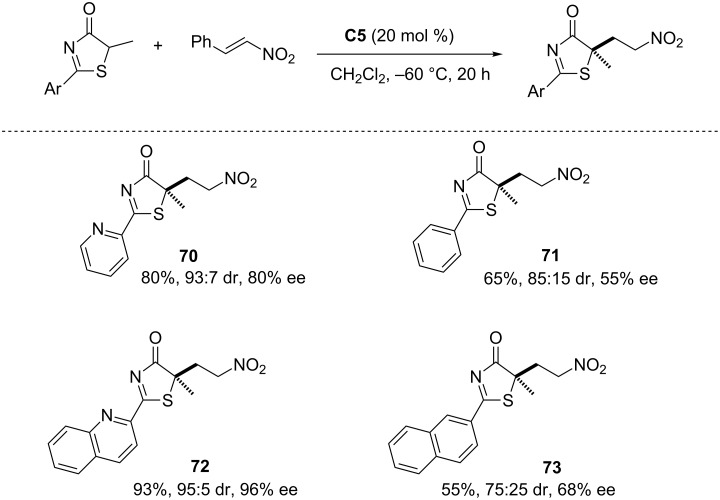
Effect of the nitrogen atom at the aromatic substituent of the thiazolone on yield and stereoselectivity in the Michael addition to nitrostyrene [85].

On the other hand, experiments carried out with pyridyl and quinoylthiazolone substrates reveal that in these cases selectivity is higher than with the phenyl and 2-naphthylthiazolones, respectively (Scheme 12, compound **70** versus **71** and **72** versus **73**). On this basis, the authors propose a bifunctional way of action of the catalyst, wherein the pyridine/quinoline nitrogen could coordinate to one of the free N–H hydrogen atoms of the catalyst.

**Scheme 13:**
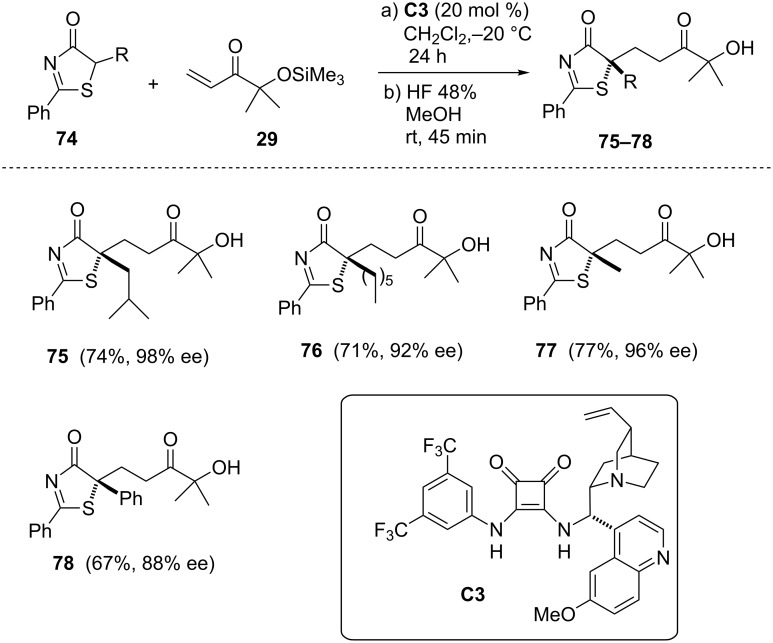
Michael addition reaction of thiazol-4(5*H*)ones **74** to α’-silyloxyenone **29** [73].

**α’-Silyloxyenones as acceptors.** As in the case of imidazolones, thiazolones also exhibit very poor reactivity in the Michael addition to methyl and *tert*-butyl acrylates. This problem could be circumvented by using α’-silyloxyenones as acrylate surrogates [73]. After catalyst screening and optimization of the reaction conditions the authors found that thiazolones **74** bearing either short, large or ramified alkyl chains at the heterocycle afforded the corresponding Michael adducts in good yields and excellent enantioselectivity in the presence of 20 mol % of catalyst **C3** (Scheme 13). Initial attempts to carry out the reactions with the α’-hydroxyenone provided the adducts in very poor enantioselectivity. This is a particular and interesting characteristic of these templates because the α-hydroxy group can be transformed into other oxy derivatives to better adapt to the most suitable substrate/catalyst interaction.

**Scheme 14:**
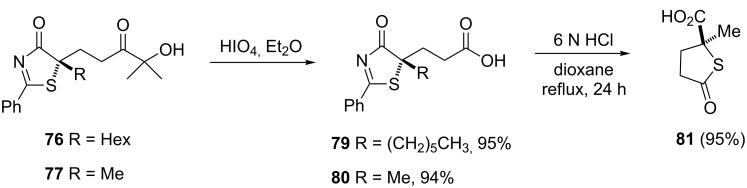
Elaboration of the thiazolone Michael adducts [73].

In addition, adducts **76** and **77** were easily transformed into the corresponding carboxylic acids **79** and **80** by treatment with periodic acid (Scheme 14) and thiolactone **81** by simple ring opening of the latter under mild acidic conditions.

**Scheme 15:**
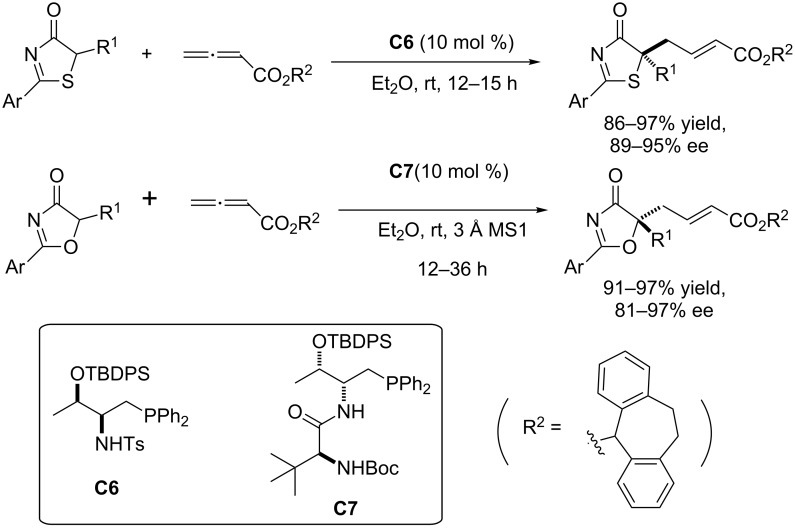
Enantioselective γ-addition of oxazol-4(5*H*)-ones and thiazol-4(5*H*)-ones to allenoates promoted by **C6**/**C7** [36].

**Phosphine-catalyzed γ-addition to allenoates and alkynoates.** Chiral phosphine-mediated nucleophilic catalysis has attracted considerable attention in the recent years [98–101]. However, few examples of phosphine-mediated γ-addition reactions have been reported. Pioneering studies on γ-additions of pronucleophiles to allenoates or alkynoates were first published by the groups of Trost [102–104] and Lu [105] in the 1990s. However, asymmetric variants were not reported until more than a decade later. The group of Fu has recently described the enantioselective γ-addition of oxygen [106–107], carbon [108], sulfur [109], and nitrogen [110] pronucleophiles to γ-substituted allenoates and/or alkynoates in the presence of a *C*_2_-symmetric chiral phosphine catalyst. Although γ-substituted allenes have been employed in many phosphine-mediated γ-additions, to date there was virtually no progress on the use of prochiral pronucleophiles in phosphine-mediated γ-additions until the group of Lu published in 2014 the addition of 3-substitued oxindoles [111]. More recently, Lu together with Lan also demonstrated the efficiency of oxazol-4(5*H*)-ones and thiazol-4(5*H*)-ones as pronucleophiles in the highly enantioselective phosphine-catalyzed asymmetric γ-addition to allenoates, which after elaboration of the resulting adducts, affords tertiary thioethers and alcohols [36] (Scheme 15).

Starting from readily available L-valine and L-threonine the authors first synthesized different phosphine catalysts and then screened in the γ-addition reaction of both, thiazol-4(5*H*)-ones and oxazol-4(5*H*)-ones. They found that whilst catalyst **C6** was the best for the reaction with thiazol-4(5*H*)-ones, in the case of oxazol-4(5*H*)-ones, however, best results were provided by catalyst **C7**. The reaction conditions were further optimized by varying both, the solvent and the ester moiety of the allenoate. Examination of different allenoates revealed that the dibenzosuberyl ester provided the best results.

**Scheme 16:**
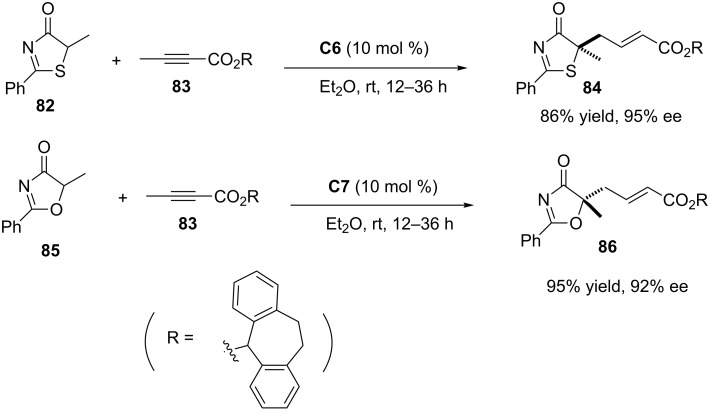
Enantioselective γ-addition of thiazol-4(5*H*)-ones and oxazol-4(5*H*)-ones to alkynoate **83** promoted by **C6**/**C7** [36].

The scope of these catalysts was also proven in the reaction with alkyne substrates. For instance, both thiazol-4(5*H*)-one **82** and oxazol-4(5*H*)-one **85** react with alkynoate **83** to provide adducts **84** and **86**, respectively, with equal efficiency than allenoates (Scheme 16).

**Scheme 17:**
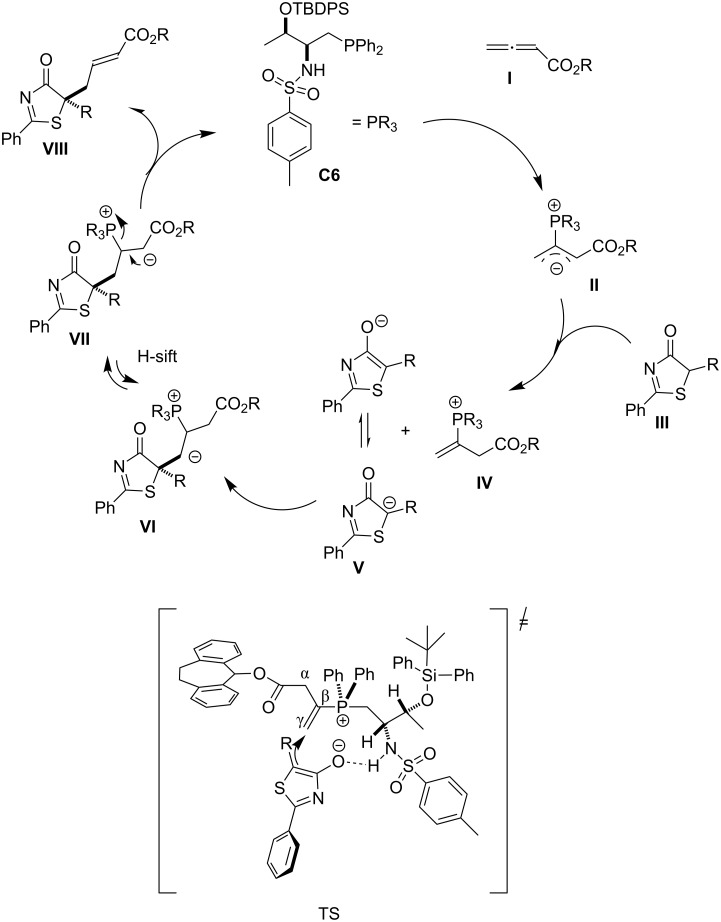
Proposed mechanism for the **C6**-catalyzed γ-addition of thiazol-4(5*H*)-one to allenoates. Adapted from [36], copyright 2015 The Royal Society of Chemistry.

The authors propose the mechanism outlined in Scheme 17 on the basis of some DFT calculations. Accordingly, the first step is the γ-addition of the phosphine to the allenoate to produce intermediate **II**. Then this basic intermediate is proposed to deprotonate the starting thiazolone thus providing enolate **V**, which subsequently adds to the γ-carbon of **IV** to afford intermediate **VI**. After proton shift, intermediate **VI** becomes intermediate **VII** and this eliminates the phosphine catalyst, which enters a new catalytic cycle, while providing the final product **VIII**. The authors propose a bifunctional behavior of the catalyst, wherein a hydrogen bonding interaction between the sulfonamide N–H and the thiazolone enolate controls its addition to the C–C double bond, which is the key step for asymmetric induction.

**Scheme 18:**
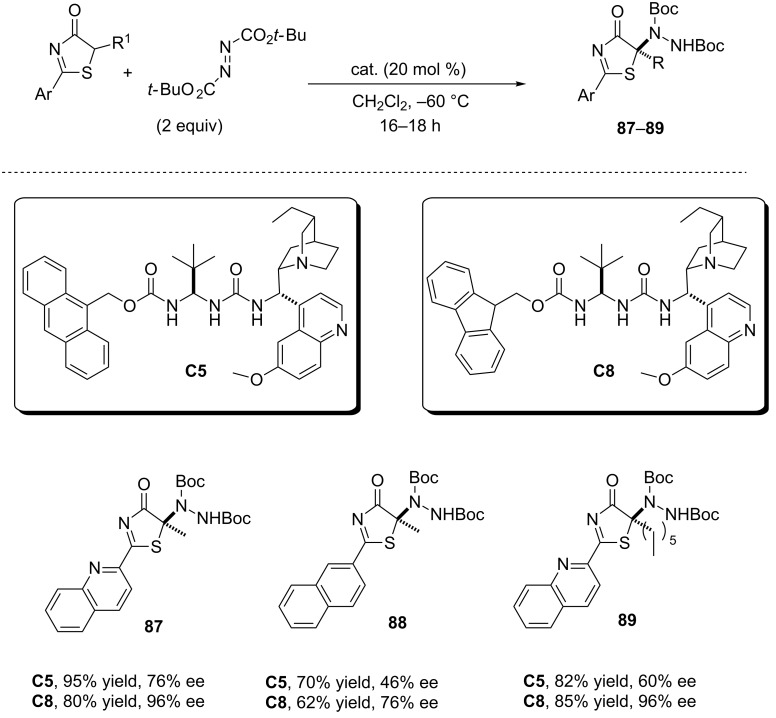
Catalytic enantioselective α-amination of thiazolones promoted by ureidopeptide like catalysts **C5** and **C8** [85].

**2.2.2 α-Amination reactions.** Thiazolones **2** have also been investigated in the α-amination reaction with *tert*-butyl azodicarboxylate in the presence of the ureidopeptide like catalysts **C5** and **C8** (Scheme 18) [85]. In these cases better enantioselectivity was observed with catalyst **C8**, and thiazolones bearing the quinoyl moiety provided once again better stereochemical results than 2-naphthylthiazolones.

**Scheme 19:**
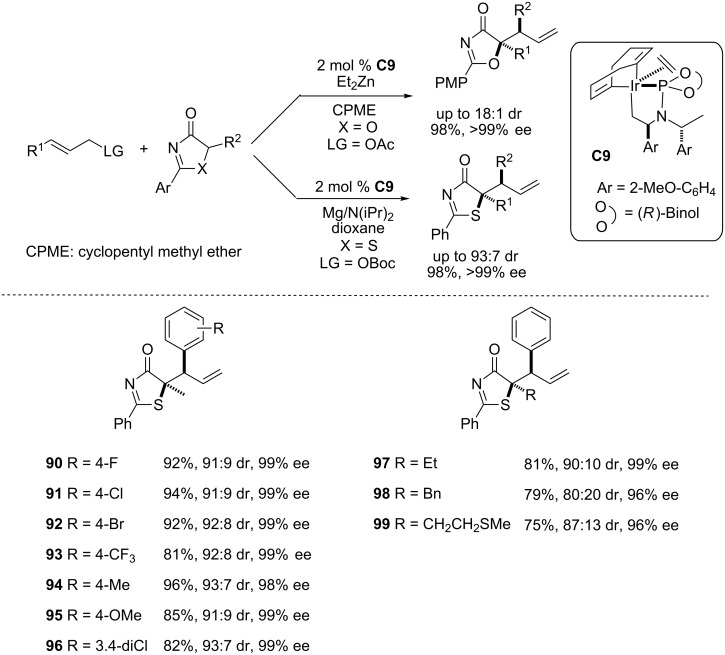
Iridium-catalized asymmetric allyllation of substituted oxazol-4(5*H*)-ones and thiazol-4(5*H*)-ones promoted by **C9**. PMP = *p*-methoxyphenyl [42].

**2.2.3 Iridium-catalyzed allylic substitution reactions.** Allylic substitution reactions catalyzed by cyclometalated iridium phosphoramidite complexes constitute a powerful tool for the construction of C–C and C–heteroatom bonds [112] and have been used in many synthetic applications [113]. However one of the main limitations in the area is the lack of highly diastereo- and enantioselective protocols. In this context, particularly difficult has been to achieve high diastereoselectivity, and only a very narrow range of nucleophiles have been reported to be efficient in this regard (α,α-disubstituted aldehydes [114] and β-ketoesters [115] among others). With the aim of expanding the nucleophile scope of this transformation, in 2014 Hartwig et al. reported a highly diastereoselective iridium-catalyzed allylation substitution reaction of oxazol-4(5*H*)-ones and thiazol-4(5*H*)-ones to afford enantioenriched tertiary alcohols and thioethers (Scheme 19) [42]. In this case the diastereoselectivity is controlled by cations, in contrast to most of the described protocols wherein it is modulated by anions.

The authors found that whilst the best results for allylation of oxazol-4-(5*H*)-ones were achieved from zinc enolates, thiazol-4-(5*H*)-ones produced the best outcome when the magnesium enolate was used. After optimization it was shown that the reaction was efficient with different cinnamyl *tert*-butyl carbonates (Scheme 19) to afford compounds **90**–**99**in general with good yields, diastereomeric ratios and enantioselectivities. Aliphatic carbonates are also good substrates for this reaction but the corresponding adducts are obtained in lower diastereoselectivity. This work demonstrates that the selectivity of the reaction is dictated by the metallacyclic iridium complex and that the nature of the enolate counterion is significant to this respect.

## Conclusion

In this short review we have summarized the published examples of the utility of 2-thio-1*H*-imidazol-4(5*H*)-ones and thiazol-4(5*H*)-ones as pronucleophiles in asymmetric catalytic reactions. The results show that they are efficient substrates in reactions which involve the creation of a tetrasubstituted stereogenic center. Further elaboration of adducts coming from these reactions gives access to *N*-substitued α-amino acids in the case of imidazolones and to tertiary thiols and thioethers in the case of thiazolones. In the future, other Brønsted base or bifunctional catalyst promoted reactions with these compounds can be envisaged.
